# The relationship between fatigue and psychiatric disorders: Evidence for the concept of neurasthenia^☆^

**DOI:** 10.1016/j.jpsychores.2008.12.007

**Published:** 2009-05

**Authors:** Samuel B. Harvey, Simon Wessely, Diana Kuh, Matthew Hotopf

**Affiliations:** a Institute of Psychiatry, King’s College London, London, UK; b MRC Unit for Lifelong Health and Ageing, Department of Epidemiology and Public Health, Royal Free and UCL Medical School, London, UK

**Keywords:** Fatigue, Chronic fatigue, Neurasthenia, Psychiatric disorder, Mental disorder, Exercise, Body weight

## Abstract

**Objective:**

Fatigue and psychiatric disorders frequently occur comorbidly and share similar phenomenological features. There has been debate as to whether chronic fatigue, or neurasthenia, should be considered an independent syndrome distinct from psychiatric disorders. We aimed to establish whether persistent fatigue can occur independently from psychiatric disorders and to test the hypothesis that fatigue without comorbid psychiatric symptoms has unique premorbid risk factors. We also aimed to investigate the psychological outcome of any individuals with fatigue.

**Methods:**

The MRC National Survey of Health and Development was used to prospectively follow 5362 participants from birth. A sample of nonfatigued individuals without psychiatric disorder was selected at age 36 and followed until age 43 years (*n*=2714). At age 43, the presence of new onset fatigue and/or psychiatric disorder was assessed. Information on a number of potential premorbid risk factors was collected between ages 0 and 36 years. Individuals with fatigue but no comorbid psychiatric disorder were then followed up at age 53 years.

**Results:**

At age 43 years, 201 (7.4%) participants reported significant levels of new onset fatigue in the absence of comorbid psychiatric disorder. Despite the absence of case level psychiatric disorder, these individuals did report increased levels of some psychological symptoms. Excessive childhood energy (adjusted OR 2.63, 95% CI 1.55–4.48, *P*<.001) and being overweight at age 36 (adjusted OR 1.62, 95% CI 1.05–2.49, *P*=.03) were specific risk factors for fatigue without psychiatric disorder but not fatigue with comorbid psychiatric illness. Neuroticism was a risk factor for fatigue both with and without comorbid psychiatric disorder. Negative life events and a family history of psychiatric illness were only risk factors for fatigue when it occurred comorbidly with psychiatric illness.

**Conclusions:**

A significant proportion of the adult population will suffer from fatigue without comorbid psychiatric disorder. While fatigue and psychiatric disorders share some risk factors, excessive energy in childhood and being overweight as an adult appear to be specific risk factors for fatigue. Our results confirm the significant overlap between fatigue and psychiatric disorders, while also providing evidence for neurasthenia as a separate diagnosis.

## Introduction

Fatigue is one of the most common symptoms encountered in medical consultations. Both primary care and community studies have found that around one quarter of all people report recent problems with fatigue [1–3]. The subjective nature of fatigue has made it difficult to define or conceptualize, although most would consider it to be an extreme and persistent form of mental and/or physical tiredness, weakness, or exhaustion [4].

Previous cross-sectional studies have demonstrated a close association between chronic fatigue and psychiatric disorders [3,5,6]. Up to two thirds of people reporting fatigue lasting longer than 6 months will also be suffering from a comorbid psychiatric disorder [7]. Prospective studies have also shown that psychiatric disorders increase the risk of later chronic fatigue [8]. While there is considerable overlap in the phenomenology of fatigue and common psychiatric disorders such as depression or anxiety, there are also some important differences. In contrast to most depressed patients, people with chronic fatigue tend to experience very little self-blame or lowered self-esteem and will often attribute their symptoms to external causes [9]. Despite this, the phenomenological similarities, together with the high rates of comorbidity, have led some authors to suggest that there is little to be gained from distinguishing medically unexplained fatigue from psychiatric disorders [10]. Others have argued that fatigue is distinguishable from depression, anxiety, or other forms of psychological distress and therefore should be considered as a separate diagnostic category [11]. The fatigue syndrome neurasthenia has been retained as a separate diagnosis within the *ICD-10* classification system [12], although it is no longer included as a separate diagnosis within the influential *DSM-IV* classification, where it is only included as undifferentiated somatoform disorder [13]. *ICD-10* classification defines neurasthenia as persistent and distressing complaints of increased fatigue after mental effort, or persistent and distressing complaints of body weakness and exhaustion after minimal effort, with accompanying somatic symptoms in the absence of a depressive illness or anxiety disorder [12].

Van der Linden et al. [14] attempted to demonstrate the existence of an independent ‘pure’ fatigue state by prospectively following 1177 patients recruited from primary care, with three separate measurements of fatigue and psychiatric disorder. While they replicated previous findings of a strong correlation between fatigue and psychiatric morbidity, they also identified a group of patients with persistent, independent fatigue, which was not associated with any increase in psychological morbidity. A similar group of individuals with fatigue, but no psychiatric comorbidity, was identified in the Baltimore sample of the Epidemiological Catchment Area study, although in this study fatigue was found to be an independent predictor of later psychiatric disorder [15]. An alternative epidemiological method of examining the differences between fatigue and psychiatric disorders is to consider shared vs. independent risk factors. Hickie et al. [16] have previously used twin studies to demonstrate that while fatigue and psychological distress share some common genetic factors, fatigue has a number of independent genetic and environmental risk factors. While previous research has identified various risk factors for both fatigue and psychiatric disorders, to our knowledge, no studies have ever attempted to identify which of these risk factors are shared and which, if any, may be independent risk factors for fatigue, but not psychiatric disorders. Possible candidates include factors previously identified as increasing the risk of fatigue, regardless of possible psychiatric comorbidity. These include being female [8,17], emotional instability (neuroticism) [18], limiting longstanding medical conditions in childhood [19], premorbid sedentary and overactive behavior [19–21], and negative life events [22].

In this study, we used a large prospective British birth cohort to investigate the temporal associations between fatigue, psychiatric disorders, and a number of potential premorbid risk factors. The large size of this cohort and the detailed information collected over the first 53 years of each participant's life provided us with a unique opportunity to test a number of related hypotheses. We aimed (1) to confirm previous observations that a substantial proportion of individuals with persistent fatigue do not suffer from comorbid psychiatric disorder; (2) to investigate premorbid risk factors for both fatigue and psychiatric disorders to establish which factors are shared and which are unique to fatigue; and (3) to examine the psychological outcome of those individuals with fatigue without any comorbid psychiatric disorder.

## Methods

### Sample

The Medical Research Council's National Survey of Health and Development is based on a random social class stratified sample of 5362 participants selected from all single, legitimate births occurring in England, Wales, and Scotland in 1 week of March 1946. This sample has been prospectively followed with over 20 separate data collections up to the age of 53 years. The sampling procedure and follow-up have been described in detail elsewhere [23].

### Fatigue and psychiatric disorder

Fatigue was assessed at two separate time points in each participant's adult life, at age 36 and 43 years. Psychiatric assessments occurred at ages 36, 43, and 53 years. At age 36 years, participants were visited at home and the 40-item version of the Present State Examination (PSE) [24] was administered by a trained nurse interviewer [25]. Psychiatric disorder was defined at a threshold level of ≥5 on the Index of Definition score [26]. The PSE contains questions about fatigue, resulting in a subscale score for fatigue and lack of energy. This subscale is calculated from the question, “Do you seem to be slowed down in your movements, or have too little energy recently? How much has it affected you?” At age 43 years, participants were again visited at home and interviewed by trained nurses using the Psychiatric Symptom Frequency scale (PSF) [27]. The PSF is an 18-item scale measuring psychiatric symptoms, particularly of depression and anxiety, over the last year. A cut-off score of ≥23 was used to define psychiatric disorder [27]. As part of the PSF, participants were asked, “Over the last year have there been days when you tired out very easily?” with six options ranging from *never* to *always*. Each of these options was defined both in terms of the frequency and duration of fatigue. Participants who selected either ‘quite often,’ ‘very often,’ or ‘always’ were classed as suffering from significant fatigue. This grouping ensured that only participants whose fatigue was present for longer than 1 month or present for 12 months on at least 2 days each week were included in the fatigued group. The 28-question version of the General Health Questionnaire (GHQ) was completed by participants at age 53 years, with probable psychiatric disorder being identified by a total GHQ score (using GHQ scoring method) of ≥five [28].

In order to be confident about the timing of onset for any fatigue or psychiatric disorder, the assessment at age 36 years was used to define a ‘healthy’ cohort without any significant fatigue or psychiatric disorder. The subsequent assessment at age 43 was then used to define four separate groups: (1) no fatigue or psychiatric disorder, (2) fatigue without psychiatric disorder, (3) fatigue with comorbid psychiatric disorder, and (4) psychiatric disorder without fatigue.

### Premorbid risk factors

Information on a number of potential premorbid risk factors was collected during various waves of data collection between ages 0 and 36 years. The premorbid factors investigated were all selected on the basis of previous research suggesting they may be risk factors for either fatigue or psychiatric disorder. All hospitalizations during childhood were recorded with the relevant hospital being contacted for details of the admission. This allowed a variable to be generated which identified any childhood physical disease that occurred between birth and age 15 years which led to at least 3 weeks of hospitalization [29,30]. Conditions such as mental handicap, psychiatric disorder, and ill-defined conditions were excluded from this group. At age 13 years, school teachers were asked to rate a range of participant's attributes, including their level of energy. Each participant's energy levels were rated as either ‘normal,’ ‘always tired,’ or ‘extremely energetic.’ Both participants and their mothers were also asked about any family history of “nervous or emotional trouble or depression” (at ages 15, 36, and 43 years) allowing a family history of psychiatric disorder to be identified [8].

At age 26 years, individuals completed the short form of the Maudsley Personality Inventory [31], which provides a measure of personality along the neuroticism and extroversion spectrums. At age 36 years, participants were asked about the frequency and duration of their involvement in a range of different forms of physical activity. A total of 27 different sports and recreational activities were enquired about. Based on their responses participants were grouped into three groups: inactive, less active, and most active. The details and rationale of the criteria used in defining these groups have been published previously [32]. Participants' heights and weights were also measured by the nursing staff during the home visit at age 36 years allowing their body mass index (BMI) to be calculated. Negative life events were enquired about during the structured interview at age 36 years. Participants were asked whether they had experienced eight different possible life events over the preceding year. Participants were asked about events including deaths among their close friends or family, serious illness, divorce, separation, crises or disappointments in their work, burglary, robbery, injuries, other crises or worries about close friends or family. It was recorded whether each event had occurred (score=1) or not (score=0) allowing a continuous aggregated life-event score to be constructed [33].

Sociodemographic factors were also an important consideration. Females are known to have increased rates of both fatigue and psychiatric disorders [17,34]. Previous studies have also suggested that gender and socioeconomic status may have an important confounding role in the relationship between fatigue and psychiatric disorders [7]. Sociodemographic details including gender, participants' social class (at age 36 years), and participants' educational level were obtained. Social class was derived from the participant's occupation using the Registrar General's classification [35], while highest education level achieved by age 26 years was coded using the Burnham classification [36].

### Statistical analysis

**Fig. 1:**
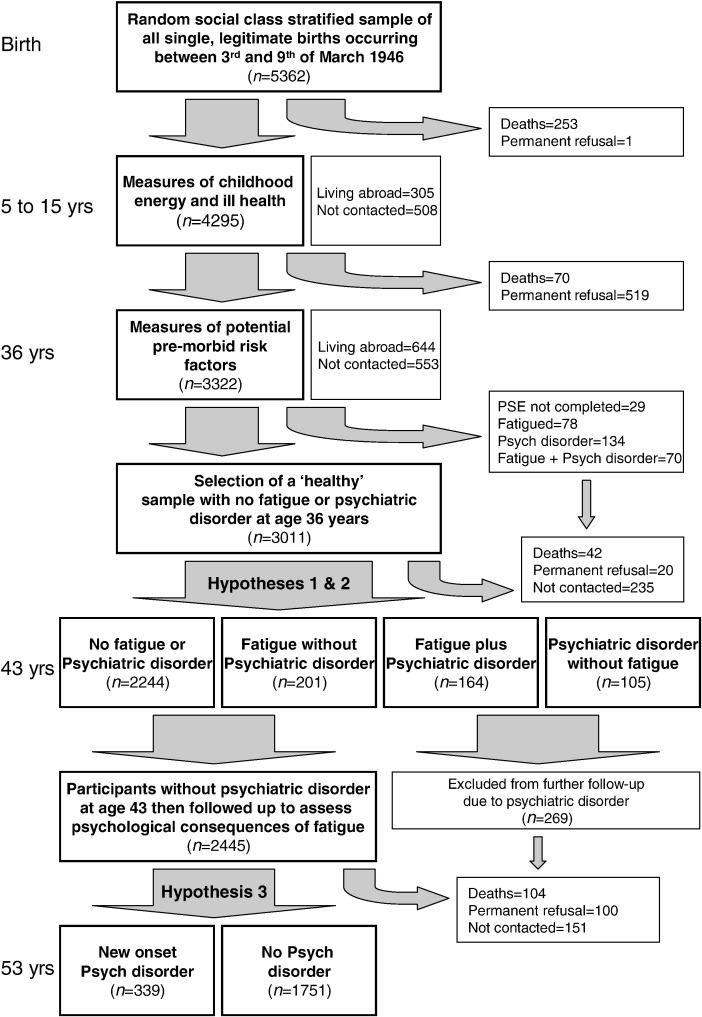
Flowchart describing analysis strategy and loss to follow-up.

Statistical analysis was performed using STATA computer software [37]. In order to test Hypotheses 1 and 2, a ‘healthy’ population (*n*=3011) without any evidence of fatigue or psychiatric disorder from the PSE at age 36 years was selected and followed up until the age of 43 years. Participants who did not complete the PSF assessment at age 43 years (*n*=296, 9.8%) were excluded leaving 2714 for analysis. Chi-squared and *t* tests were used to identify any important differences between those included in the analyses and those lost to follow-up. Multinomial (polytomous) logistic regression was then used to investigate the associations between various premorbid factors and the four outcome groups defined by the presence or absence of fatigue and psychiatric disorder at 43 years (Hypothesis 2). All analyses were adjusted for gender, social class, and education level. We then focused on the psychological outcome of participants with fatigue without any comorbid psychiatric disorder at age 43 years (Hypothesis 3). For this final analysis, participants with evidence of psychiatric disorder at age 43 were excluded (*n*=269), leaving a sample of 2445 participants. Individuals with different levels of fatigue, but no comorbid psychiatric illness, were followed up to the age of 53 years for ‘new onset’ psychiatric disorder. Logistic regression was used to provide estimates for the effect size of any association, with adjustments for possible confounding factors. The overall analysis plan is demonstrated in Fig. 1.

### Ethical approval

Ethical approval was given by the London area multicenter research ethics committee for the data collection at age 53 years. Cohort members gave informed consent for each assessment. Ethical approval and consent procedures at earlier ages conformed to contemporary best practice.

## Results

Of the 5362 individuals selected at birth, 3322 were successfully followed up to the age of 36 years. After exclusion of those who died (*n*=323), were living abroad (*n*=644), or had permanently refused to participate (*n*=520), this represented a follow-up rate of 86%. A total of 3293 of these individuals (99.1%) completed a PSE examination at age 36 years. Two hundred and four (6.2%) demonstrated a likely psychiatric disorder, and 148 (4.5%) scored highly on the fatigue and lack of energy PSE subscale (with 70 individuals suffering from both psychiatric disorder and fatigue). These 282 individuals were excluded to create a sample of 3011 ‘healthy’ individuals without any evidence of fatigue or psychiatric disorder. A total of 2715 (90.2%) of these ‘healthy’ individuals were successfully followed up to age 43, where the presence of new onset fatigue and psychiatric disorder was assessed. A flow diagram demonstrating this and the reasons for any loss to follow-up is provided in Fig. 1. There was no difference between those assessed at age 43 and those lost to follow-up in terms of gender, social class, or personality measures. However, individuals with lower levels of education (*P*<.001), no family history of psychiatric disorder (*P*=.003), less negative life events (*P*=.05), and who were less energetic as children (*P*=.02) were more likely to be lost to follow-up prior to the age of 43 years.

The characteristics of the 2714 individuals followed up to age 43 years are described in Table 1. At age 43 years, 365 (13.4%) of this previously nonfatigued sample reported significant levels of fatigue (fatigue with a duration of >1 month or present for 12 months on at least 2 days each week). Of the 365 fatigued individuals, 164 (44.9%) were suffering from comorbid psychiatric disorder. Two hundred and one participants (55.1% of the fatigued group and 7.4% of the entire sample) had fatigue without any comorbid psychiatric disorder. Despite this group of fatigued individuals not meeting the case definition for psychiatric disorder, they did report significantly increased levels of many associated symptoms including feeling mentally tense, low appetite, difficulty sleeping, low mood, and impaired concentration (*P*≤.001). However, they did not report increased levels of anxiety symptoms (panic, situational anxiety, or fear of becoming ill) and only 28 (13.9%) reported significant levels of low mood.

Table 2 demonstrates the associations between the different combinations of fatigue and psychiatric disorder and a number of potential premorbid risk factors. Neuroticism was associated with an increased risk of both fatigue and psychiatric disorder in all possible combinations. The effect size was largest for comorbid fatigue and psychiatric disorder, with an adjusted odds ratio (OR) of 2.13 (95% CI 1.42–3.17, *P*<.001). Negative life events at age 36 years increased the risk of psychiatric disorder, both with and without fatigue, at age 43 years. Negative life events did not increase the risk of fatigue in the absence of comorbid psychiatric disorder. Both a family history of psychiatric disorder and physical inactivity at age 36 were risk factors for fatigue, but again only when the fatigue was comorbid with psychiatric disorder. Participants whose teachers had reported as being extremely energetic at age 13 years were significantly more likely (adjusted OR 2.20, 95% CI 1.33–3.65, *P*=.002) to report fatigue without comorbid psychiatric disorder at age 43 years. Energetic children were not at increased risk of fatigue comorbid with psychiatric disorder. Participants who were overweight at age 36 were also at increased risk of fatigue in the absence of psychiatric disorder at age 43 years (adjusted OR 1.56, 95% CI 1.07–2.26, *P*=.02). While obese individuals did not appear to be at increased risk, there was moderate evidence of a positive linear association between BMI and risk of later fatigue without comorbid psychiatric disorder (*P*=.05). There was no evidence of any association between chronic illness in childhood or extraversion and the risk of later fatigue or psychiatric disorder.

A final multivariable model was created which included all significant premorbid risk factors together with gender, social class, and education. Females were at increased risk for both fatigue and psychiatric disorder. Being described as extremely energetic at age 13 (adjusted OR 2.63, 95% CI 1.55–4.48, *P*<.001) and being overweight at age 36 (adjusted OR 1.62, 95% CI 1.05–2.49, *P*=.03) were both independent predictors of fatigue without psychiatric disorder. Neuroticism as a categorical variable was not an independent predictor of fatigue without comorbid psychiatric disorder, although when considered as a continuous variable in a post hoc analysis an independent effect was seen (*P*=.04). Those with fatigue comorbid with psychiatric disorder were more likely to have a family history of psychiatric disorder (adjusted OR 1.94, 95% CI 1.26–2.98, *P*=.003), less likely to have been physically active (adjusted OR 0.51, 95% CI 0.31–0.86, *P*=.01), and more likely to score highly for neuroticism (adjusted OR 1.84, 95% CI 1.18–2.88, *P*=.07). The impact of increased negative life events on the risk of future comorbid fatigue and depression was reduced to borderline significance (adjusted OR 1.53, 95% CI 0.99–2.37, *P*=.06).

In order to examine the psychological outcome of fatigue without any comorbid psychiatric disorder, we then focused on the 2445 participants who did not have any evidence of psychiatric disorder at either age 36 or 43 years. The reasons for any exclusions and loss to follow-up are demonstrated in Fig. 1. The selected group of participants without psychiatric disorder contained individuals with a range of fatigue. They were divided into those with no fatigue, those with mild fatigue (up to 1-month duration or once or twice a week for the last 12 months), and those with more severe fatigue (more than 1-month duration or more often than twice a week for the last 12 months). A total of 937 (38.3%) of the participants reported mild fatigue, while 201 (8.2%) individuals described more severe fatigue. As demonstrated in Table 3, both mild and severe fatigue (without comorbid psychiatric illness) were associated with increased risk of later psychiatric disorder. There did not appear to be a linear dose–response effect, with the effect size of the increased risk being similar for severe and mild fatigue. Likelihood ratio tests confirmed that a linear model was not appropriate. The increased risk of later psychiatric illness remained after adjustment for social demographics (gender, social class, and education) and other potential confounders (personality, negative life events, family history of psychiatric disorder, childhood illness, energy levels as a child, physical activity as an adult, and BMI).

## Discussion

### Key findings

This large, population-based prospective study has provided further evidence of the considerable comorbidity between fatigue and psychiatric disorders. We have, however, also identified a group of patients who suffer from significant fatigue without comorbid psychiatric disorder. We have been able to demonstrate for the first time that fatigue with and without comorbid psychiatric disorder have different sets of premorbid risk factors. Despite the differences identified, we found those suffering from fatigue without any comorbid psychiatric disorder had increased levels of some psychological symptoms and remained at increased risk of later psychiatric disorder, regardless of the severity of fatigue.

These results should not be misinterpreted as suggesting psychological factors are not of vital importance in the etiology and understanding of chronic fatigue. Our results should also not be interpreted as suggesting that individuals with ‘pure’ fatigue have a more genuine or real disorder than those with fatigue and comorbid psychiatric illness. A psychological understanding of fatigue, and in many cases psychologically informed treatments, remains important regardless of whether other comorbid psychiatric disorders are present. What our results do demonstrate is the way in which fatigue presents and in particular whether fatigue co-occurs with psychiatric disorders seems to depend to some extent on various premorbid factors.

### Evidence for the concept of neurasthenia

The diagnosis of fatigue syndromes has often been controversial with considerable discussion about whether fatigue can be considered separately from psychiatric disorders [38]. Within our study, over 7% of the adults aged 43 years reported significant levels of new onset (since age 36 years) fatigue without any comorbid psychiatric disorder. Other authors have estimated the prevalence of fatigue without psychiatric comorbidity to be around 7% [39]. The exclusion of individuals with fatigue at age 36 years in our study means we were only capturing new (incident) cases and therefore we cannot provide a prevalence estimate. However, taken together, these results show that fatigue without psychiatric disorder is sufficiently common to make it a significant clinical issue. In contrast to both Van der Linden et al. [14] and Hickie et al. [39], we found that individuals with fatigue in the absence of comorbid psychiatric disorder were at increased risk of later psychiatric illness. This is perhaps due to our larger sample size and longer follow-up period. This increased risk remained even after controlling for the effects of shared risk factors and other possible confounders.

Chronic fatigue syndrome (CFS), as defined by the Centers for Disease Control and Prevention, requires an individual to have at least 6 months of unexplained fatigue with a substantial reduction in function together with at least four associated somatic or cognitive symptoms [40]. Previous studies have shown a strong association between CFS and psychiatric disorders [8]. The diagnosis of neurasthenia, as defined by the *ICD-10* classification, is similar, although it does not stipulate the duration of fatigue and requires only two associated somatic symptoms [12]. In addition, the current definition of neurasthenia specifically excludes any individual meeting diagnostic criteria for depression or anxiety. Within our study, we have identified a group of individuals with significant fatigue in the absence of comorbid psychiatric disorder. This definition of a fatigue state is very similar to neurasthenia (although without any assessment of accompanying somatic symptoms), but is significantly different from the current criteria for diagnosing CFS. The exact criteria used to define syndromes such as neurasthenia and CFS are, to some degree, arbitrary [38,41]. However, the differences between these definitions are important to consider, particularly when investigating the relationship between fatigue and psychiatric disorders. Previous studies have shown that as additional somatic symptoms are added to the diagnostic criteria for fatigue syndromes, the association with psychiatric disorders increases [42,43]. It is therefore likely that the relative incidence of comorbid fatigue and psychiatric disorder would be significantly higher if associated somatic symptoms or disability was required for a diagnosis. As such, our results cannot be directly extrapolated to those diagnosed with CFS.

The observation that a positive family history, neuroticism, and negative life events increase the risk of subsequent psychiatric disorders is not new [44–47]. Previous studies have additionally suggested that each of these factors increases the risk of fatigue [8,18,22]. In this study, we have confirmed that negative life events and a family history of psychiatric disorder are risk factors for later fatigue, but only fatigue which is comorbid with psychiatric disorder. Neither of these factors increased the risk of later fatigue without comorbid psychiatric disorder. In contrast, neuroticism increased the risk of fatigue both with and without comorbid psychiatric disorder. The longitudinal association between neurotic personality traits and depression is thought to reflect shared genetic vulnerability [48]. Neuroticism may also represent a shared genetic risk for both fatigue and psychiatric disorder.

The observation that fatigue has different risk factors compared to fatigue comorbid with psychiatric disorders is consistent with the predictions from twin studies [16,49]. We identified two premorbid factors which appeared to act as risk factors for fatigue without comorbid psychiatric disorder, but not for fatigue comorbid with psychiatric disorder: extreme childhood energy and a BMI in the overweight range. Excessive childhood energy has previously been associated with an increased risk of self-reported adult chronic fatigue syndrome [20] and has recently been reported to be associated with severe fatigue in adolescents [21]. The reasons for these apparently contradictory associations are not yet clear, although one possible explanation is that overactivity in childhood is associated with the development of personality traits that are not captured by standard personality assessments. One such trait may be the ‘hyperactive’ or ‘action-prone’ personality proposed by Van Houdenhove et al. [50,51]. They suggest that these individuals have an orientation toward direct action and often use overactivity as a coping strategy or as a way of maintaining their self-esteem [51]. This may make them more prone to developing physical complaints such as fatigue following a period of incapacity or when their increasing age restricts their activity [52]. The observation of increased risk of fatigue among overweight adults, and the lack of any association with adult levels of physical activity, suggests these individuals may have already had to restrict their activity by the age of 36 years.

The significant numbers of individuals suffering from fatigue without comorbid psychiatric disorder, and the identification of independent risk factors for this group, support the concept of fatigue syndromes such as neurasthenia remaining as independent diagnoses. This does not necessarily mean that fatigue with and without comorbid psychiatric disorder should be considered as separate syndromes. As Stubhaug et al. point out, “strict adherence to diagnostic criteria is in the best medical tradition, but may be an inadequate strategy for conditions that truly are multisymptomatic and general [53].” Even those individuals identified in our study as suffering from fatigue without psychiatric disorder had increased rates of psychological symptoms and had an increased risk of psychiatric disorder in the future. Psychological interventions remain the main mode of treatment for fatigue syndromes, regardless of whether diagnosable psychiatric disorders are present [54]. The acknowledgment of the diagnostic independence of fatigue syndromes may allow some patient groups to more readily accept psychologically informed interventions.

### Strengths and limitations

This study had a number of important strengths, including the large sample size, prospective collection of data, detailed information on participants, and a long follow-up period. Despite these strengths, our study does suffer from some important limitations. At age 43 years, fatigue was measured by a single question, rather than a recognized fatigue questionnaire, and fatigue was not enquired about at all at age 53 years. Despite this, the frequency of fatigue appears to match that reported in previous studies. The use of fatigue questions that were embedded within psychiatric scales is also a potential problem, although the main effect of this should have been a reduction in the probability of fatigue not comorbid with psychiatric disorder being identified. The use of different scales of fatigue at different time points is also a limitation. The PSE is interview based and covers symptoms over the last month, while the PSF is a self-report instrument which asks about symptoms over the last year [55]. To our knowledge, there has never been a direct comparison of these two scales and the fatigue subscales have never been validated. However, the wording of the fatigue questions in both scales suggests they should capture individuals who complain of fatigue in a clinical setting, and as such they should both have reasonable validity. The secondary analysis of data such as this may also convey some advantages. As neither the participants nor the researchers were aware of the specific hypothesis we have tested, both respondent and observer bias should have been reduced. The other main limitation is the lack of detailed information on physical illness. While previous research suggests diagnosed somatic illness only explains a small percent of fatigue in the general community [15], somatic symptoms are an important part of the *ICD* criteria for neurasthenia. The absence of information on such somatic symptoms means the group identified in our study as suffering from fatigue without psychiatric disorder cannot be assumed to be identical to those diagnosed with neurasthenia.

### Conclusion

Fatigue and psychiatric disorders are common problems, which often occur comorbidly. Despite this overlap, our study has provided evidence that around 7% of adults suffer from fatigue without significant psychiatric symptoms. While fatigue and psychiatric disorders share some risk factors, excessive energy in childhood and being overweight as an adult appear to be specific risk factors for fatigue. These findings provide further evidence that individuals suffering from fatigue are a very heterogeneous group. While common mental disorders, such as depression and anxiety, are important factors in many presenting with fatigue, a significant minority of fatigued individuals (with a unique risk factor profile) will suffer from fatigue without psychiatric disorder. These individuals still have increased levels of psychological symptoms and are at increased risk of future psychiatric illness. As a result, these observations should not be seen as evidence against the importance of psychological factors in the etiology and treatment of fatigue. However, they do provide further evidence for the validity of a separate neurasthenia diagnosis.

## Figures and Tables

**Table 1:** Description of the sample followed up to the age of 43 years (*n*=2714)

Variable	*n*		Frequency (percentage in parentheses)
Gender	2714	Male	1391 (51.2%)
		Female	1323 (48.8%)
Education	2593	Up to ‘O level’	1130 (43.6%)
		‘O level’ or above	1463 (56.4%)
Participant's social class (at age 36 years)	2393	I (Professional)	202 (8.4%)
		II (Intermediate)	748 (31.3%)
		III NM (Skilled—nonmanual)	506 (21.2%)
		III M (Skilled—manual)	519 (21.7%)
		IV (Partly skilled)	343 (14.3%)
		V (Unskilled)	75 (3.1%)
Family history of psychiatric disorder	2454	No	1620 (66.0%)
		Yes	834 (34.0%)
Chronic illness as child (between ages 0 and 15 years)	2714	No	2306 (85.0%)
		Yes	408 (15.0%)
Energy as a child (teacher's report at 13 years)	2358	Tired	97 (4.1%)
		Normal	2078 (88.1%)
		Extremely energetic	183 (7.8%)
Neuroticism (at age 26 years)	2510	Low levels (0–6 on MPI)†	1397 (55.7%)
		High levels (7–12 on MPI)†	1113 (44.3%)
Extraversion (at age 26 years)	2510	Low levels (0–8 on MPI)†	1422 (56.7%)
		High levels (9–12 on MPI)†	1088 (43.3%)
Level of physical activity (at age 36 years)	2712	Inactive	960 (35.4%)
		Less active	694 (25.6%)
		Most active	1058 (39.0%)
Body mass index (at age 36 years)	2692	Healthy weight (18.5–24.9)	1739 (64.6%)
		Underweight (up to 18.4)	73 (2.7%)
		Overweight (25–29.9)	730 (27.1%)
		Obese (more than 30)	150 (5.6%)
Negative life events (aged 36 years)	2654	Low levels (0–2)†	1788 (67.4%)
		High levels (3–8)†	866 (32.6%)
Fatigue and psych disorder (aged 43 years)	2714	No fatigue or psychiatric disorder	2244 (82.7%)
		Fatigue with no psychiatric disorder	201 (7.4%)
		Fatigue with psychiatric disorder	164 (6.0%)
		Psychiatric disorder with no fatigue	105 (3.9%)

‘O’ levels=Ordinary Level Exam, an examination usually taken at age 16 which is required to continue further education. MPI=Maudsley Personality Inventory.

† Scores divided around the median.

**Table 2:** Premorbid predictors of fatigue and psychiatric disorder

Premorbid variable	No fatigue or psychiatric disorder (*n*=2244)	Fatigue with no psychiatric disorder (*n*=201)	Fatigue with psychiatric disorder (*n*=164)	Psychiatric disorder with no fatigue (*n*=105)
Family history of psychiatric disorder	1.00	1.18 (0.83–1.69)	1.96⁎⁎ (1.33–2.88)	1.46 (0.93–2.28)
Chronic illness as a child (0 to 15 years)	1.00	1.21 (0.78–1.87)	1.06 (0.64–1.76)	1.25 (0.73–2.16)
Energetic child† (teacher report aged 13 years)	1.00	2.20⁎⁎ (1.33–3.65)	1.07 (0.50–2.26)	0.65 (0.23–1.81)
‘Tired’ child† (teacher report aged 13 years)	1.00	0.67 (0.24–1.90)	1.01 (0.39–2.58)	0.51 (0.12–2.12)
Neuroticism†† (at age 26 years)	1.00	1.44⁎ (1.02–2.03)	2.13⁎⁎⁎ (1.42–3.17)	1.93⁎⁎ (1.24–3.01)
Extraversion†† (at age 26 years)	1.00	1.14 (0.81–1.59)	0.85 (0.58–1.26)	0.75 (0.48–1.16)
Most physically active† (at age 36 years)	1.00	0.80 (0.55–1.17)	0.64⁎ (0.42–1.00)	1.43 (0.84–2.42)
Less physically active† (at age 36 years)	1.00	0.83 (0.54–1.26)	0.81 (0.51–1.29)	1.51 (0.86–2.64)
Obese (BMI >30)††† (at age 36 years)	1.00	0.79 (0.34–1.86)	1.43 (0.69–2.96)	0.76 (0.27–2.15)
Overweight (25<BMI<30)††† (at age 36 years)	1.00	1.56⁎ (1.07–2.26)	1.21 (0.78–1.88)	0.87 (0.53–1.44)
Negative life events†† (at age 36 years)	1.00	1.12 (0.79–1.58)	1.73⁎⁎ (1.19–2.53)	1.57⁎ (1.01–2.43)

Values shown are relative risk ratios (95% confidence interval) derived from multinomial (polytomous) logistic regression adjusted for gender, social class, and education level.

⁎ *P*<.05.

⁎⁎ *P*<.01.

⁎⁎⁎ *P*<.001.

† Compared to those with normal energy levels (age 13 years) or inactive (age 36 years).

†† Individuals with scores above the median compared to those with scores below the median.

††† Compared to individuals with a ‘healthy’ BMI (18.5–24.9).

**Table 3:** Multivariable model describing the relationship between preexisting fatigue (without comorbid psychiatric illness) and later psychiatric disorder

Fatigue at age 43 years	Number	Percent with psychiatric disorder at 53 years	Adjusted odds ratio† (95% confidence interval)	*P* value††
None	1119	11.9	1.00	
Mild (a spell of up to 1 month or once or twice a week for the last 12 months)	807	21.1	1.92 (1.40–2.64)	<.001
Severe (a spell of more than 1 month or more often than twice a week for the last 12 months)	164	22.0	1.77 (1.02–3.08)	.04

Individuals with psychiatric illness at ages 36 and 43 years have been excluded from this analysis.

† Adjusted for gender, social class, education, childhood illness, energy as a child, level of physical activity at age 36 years, BMI at age 36 years, neuroticism, extraversion, family history of psychiatric disorder, and number of negative life events.

†† Likelihood ratio tests suggest categorical model more appropriate than linear model.
